# Readmission Risk Assessment Tool (RRAT) for Decreasing 30-Day Readmission Rates in Total Joint Arthroplasty (TJA) and Predicting Readmission

**DOI:** 10.7759/cureus.35313

**Published:** 2023-02-22

**Authors:** John Dundon, Justin Koss, Kathleen Hodapp, Charmaine Lefevre, Eileen Poletick, Jay N Patel

**Affiliations:** 1 Orthopedic Surgery, Orthopedic Institute of New Jersey, Morristown, USA; 2 College of Osteopathic Medicine, Nova Southeastern University Dr. Kiran C. Patel College of Osteopathic Medicine, Davie, USA; 3 Orthopedics, Morristown Medical Center, Morristown, USA; 4 Orthopedics, Hackensack University Medical Center, Hackensack, USA

**Keywords:** total joint replacement, readmission rate, readmission risk, risk modification, value based care, total joint arthroplasty, risk optimization, bundled payments

## Abstract

Background

Total joint arthroplasty (TJA) has moved to a value-based care model that emphasizes increased quality and decreased costs. Preoperative patient selection and optimization significantly improve postoperative outcomes, improve quality, and decrease systemic costs. We introduced a readmission risk assessment tool (RRAT) previously verified in the literature at a large, private practice, multispecialty hospital to determine if implementation could improve outcomes and decrease our readmission rates.

Methods

All patients were administered the RRAT scoring tool prior to surgery. All staff was trained prior by a team consisting of multiple orthopedic surgeons, internal medicine and cardiac specialists, and anesthesiologists. If the score received by the patient was greater or equal to 4, a letter was sent immediately to the operative physician to work on optimization and a list of options for optimization was provided. No patients were expressly denied surgery.

Results

All 4912 patients from September 2017 to March 2020 were screened using the RRAT tool. A total of 228 patients had an RRAT score greater than 4 and required notification of the index surgeon. The overall readmission rate was 2.61% for all patients. We noted a readmission rate of 2.35% for those with a score of <4, 4.27% for those between 4-6, and 13.64% for those with a readmission rate >6. The odds ratio of those readmitted with an RRAT score >6 was 6.5488 (1.9080-22.4775, 95% CI). The American Society of Anesthesiologists (ASA) score and RRAT score were significantly correlated (Spearman Rho =0.324, P<0.001). Thirty-day readmission rates across the system decreased from 3.7% to 2.61% (p<0.05) when compared to the readmission rate in the year prior to the application of RRAT (September 2016 - August 2017).

Conclusion

The preoperative RRAT score is significantly correlated with 30-day readmission rates. Notification of the surgeon preoperatively of risk factors with modification options significantly lowered readmission rates in our study. Preoperative optimization leads to a decreased readmission rate and surgeon involvement is paramount to adherence.

## Introduction

Total joint arthroplasty (TJA) is one of the most effective and rapidly growing procedures. In the United States, the volume of total knee arthroplasty (TKA) procedures is expected to increase to nearly 3.5 million procedures annually by 2030 [1]. For primary total hip arthroplasty (THA), the number of annual procedures performed is expected to rise to 1.26 million by 2060 [2]. With the increasing number of procedures being performed, the associated complications and readmission rates are also expected to rise. Costs associated with TJA are expected to rise from $5 billion in 2006 to $50 billion by 2030 [3]. Many studies have been published identifying patient risk factors for readmission in TJA patients although it is uncertain whether optimization of these risk factors can lead to lower readmission rates.

Preoperative patient selection and optimization can potentially improve postoperative outcomes, improve the quality of care, and decrease costs. Several studies have aimed at determining which patients are at increased risk for postoperative complications and readmission in order to preemptively intervene and improve their modifiable risk factors [4-9]. One such preoperative assessment tool that has been developed is the readmission risk assessment tool (RRAT). The RRAT assigns points for each of several potentially modifiable medical comorbidities to assess the risk of readmission after TJA. RRAT consists of eight modifiable risk factor categories: Staphylococcus aureus (S. aureus) colonization, tobacco use, obesity (body mass index (BMI)), cardiovascular disease, venous thromboembolic disease, neurocognitive/psychological/behavioral problems, physical deconditioning, and diabetes. Scoring for each risk factor is allotted based on the potential severity of its association with readmission on a scale of 1 to 3. A BMI of >40 kg/m^2^, fasting glucose of >180 mg/dL, and nasal colonization with S. aureus were each scored 3 given their strong association with readmissions.

Boraiah et al. developed and verified the use of RRAT in the literature at a large, private practice, multispecialty hospital [9]. The purpose of our study was to use RRAT in our patient population in a systemwide, multi-hospital study in order to identify patients at higher risk for readmission and then see if RRAT implementation could help decrease TJA readmission rates. Each TJA patient across multiple hospitals was screened using RRAT during the study period. The patient’s orthopedic surgeon was then notified of their RRAT score preoperatively. Surgeons were also given a list of possible interventions to better optimize the patient’s comorbidities prior to surgery to see if implementation could improve outcomes and decrease readmission rates.

## Materials and methods

This retrospective cohort study was done at a health system consisting of five hospitals. The hospitals include a large tertiary care medical center, a suburban regional medical center, and three community hospitals. We started with the creation of a preoperative optimization team to lead in the direction and administration of the RRAT scoring and optimization. This team consisted of four orthopedic surgeons, an anesthesiologist, an internist, a cardiologist, and multiple nurse practitioners and program coordinators. The goal was to focus on the implementation of the RRAT tool, notifications to the respective orthopedic surgeons of high-risk patients, and to provide a list of optimization methods that could be offered to the patient to help lower their risk of complications and readmissions.

Between September 2017 and March 2020, all elective TJA patients were administered the RRAT scoring tool prior to surgery by a member of the nursing staff at the required total joint preoperative education seminar. All staff were trained and familiar with the RRAT screening prior to the implementation of the study. Each patient was scored using the RRAT tool shown in Table 1. If a patient received a score greater than or equal to 4, a letter was sent to their surgeon with medical optimization methods for each of the patient’s RRAT-identified modifiable risk factors. No patients were expressly denied surgery. The readmission rate after RRAT implementation was compared to the prior year (September 2016 - August 2017), before the implementation of RRAT at the same hospitals. Patient age, race, American Society of Anesthesiologists (ASA) score, and readmission rates were summarized using descriptive statistics. The rate of readmission was compared between the subject's RRAT scores.

**Table 1:** Overview of the readmission risk assessment tool (RRAT)

	Risk Factors	Points on Risk Stratification Scale
1	Staphylococcus Aureus colonization	3
2	Smoking (tobacco use)	1
3	Obesity	
	Body mass index (BMI 30-39)	1
	BMI 35-39.9	2
	BMI >40	3
4	Cardiovascular disease	1
5	Venous thromboembolic disease (VTED)	
	Has risk factors for VTED	1
	History of pulmonary embolus or deep venous thrombosis	2
6	Neurocognitive, Psychological, and Behavioral Problems	
	Neurocognitive deficits such as dementia, traumatic brain injury, active psychiatric illness, etc	1
	Score of >7 for depression on the patient health questionnaire (PHQ)-9	1
	Alcohol or chronic active narcotic dependency	2
7	Physical deconditioning	
	Comorbidities affected physical function and ambulation	1
	Non-ambulatory or needs assistance with transfers	2
8	Diabetes	
	Well-controlled	1
	Hemoglobin A1C >8	2
	Fasting blood glucose >180 mg/dl	3

All statistical analyses were performed using MINITAB v18 software (State College, PA). A P-value of less than 0.05 was considered statistically significant. Mean, standard deviation, and range were used for continuous data, and percentages were utilized for categorical data. Categorical data were compared by chi-square, Fisher exact, or Spearman rho tests, as appropriate. Readmission rates were compared using the Poisson rate test. Logistic regression was used to calculate readmission by RRAT score.

If a patient received a score greater than or equal to 4, a letter was sent to the operative physician to work on optimization. A list of options/interventions for optimization was provided based on the different categories the patient scored in. No patients were expressly denied surgery. The readmission rate was then compared to the prior year (September 2016 - August 2017) before the implementation of RRAT.

## Results

In total, 4,912 patients were screened using the RRAT tool at their preoperative total joint education seminar during the study period. Their demographic data is outlined in Tables 2, 3. A total of 537 patients had an RRAT score greater than or equal to 4 and the required optimization notices were sent to their surgeons. The overall readmission rate was 2.61% for all patients. We noted a readmission rate of 2.34% for those with a score of <4, 4.27% for those between 4 and 6, and 13.64% for those with an RRAT score >6 as outlined in Table 4. An odds ratio and binary regression with confidence intervals (CI) were calculated for those readmitted with an RRAT score between 4-6 (1.8508 (1.1572- 2.9604, 95% CI)) and those with an RRAT score of >6 (3.5383 (0.9736 - 12.8587, 95% CI)).

**Table 2:** Race demographics of patients by the readmission risk assessment tool (RRAT) score

Race	RRAT < 4	RRAT ≥ 4	All
	N= 4,375	N=537	N=4,912
American Indian or Alaskan Native	8 (0.18 %)	0 (0%)	8 (0.16%)
Asian	99 (2.26%)	10 (1.86%)	109 (2.22%)
Black or African American	276 (6.31%)	46 (8.57%)	322 (6.56%)
Other, Unknown, Multi-Racial, or Decline to Answer	275 (6.29%)	40 (7.45%)	315 (6.41%)
White	3717 (84.96%)	441 (82.12%)	4158 (84.65%)

**Table 3:** Age demographics of patients by the readmission risk assessment tool (RRAT) score

	RRAT < 4	RRAT ≥ 4	All
	N= 4,375	N=537	N=4,912
Age, mean (SD)	67.87 (10.01)	67.739 (10.162)	67.856 (10.026)
Age range	26-96	29-97	26-97

**Table 4:** Readmission rates by the readmission risk assessment tool (RRAT) score breakdown

RRAT score	Patients	Readmission Rate	p-value	Odds ratio	Confidence Interval
RRAT score <4	4375	2.34%			
RRAT score 4-6	515	4.27%	P<0.05	1.85	1.16-2.96, 95% CI
RRAT score >6	22	13.64%	P<0.05	3.54	0.97-12.86, 95%CI

**Figure 1:**
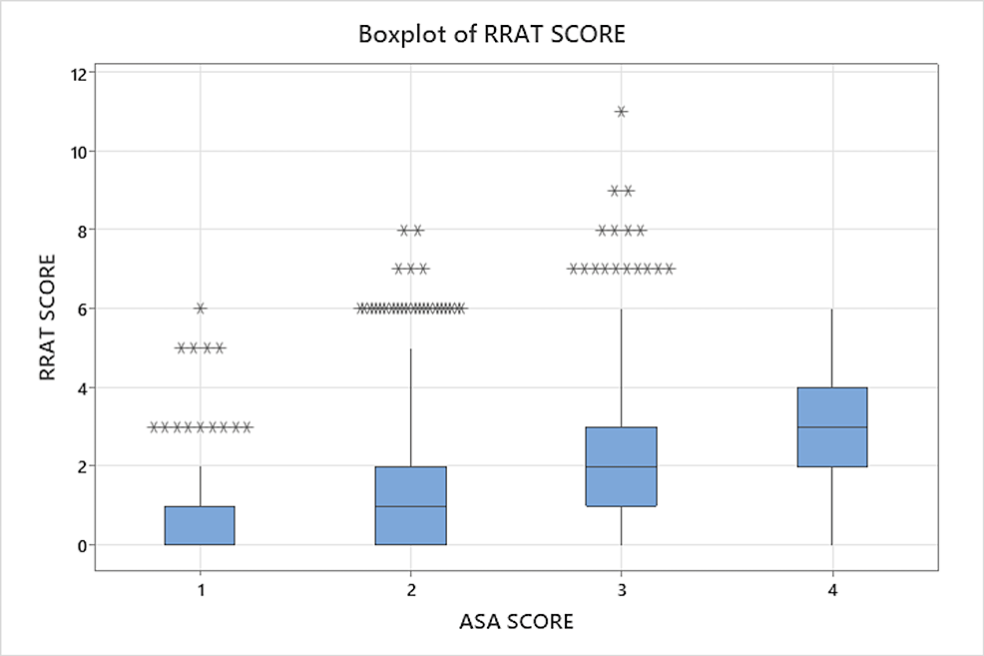
Boxplot comparing the American Society of Anesthesiologists (ASA) score to the readmission risk assessment tool (RRAT) score

ASA scores were also recorded for each patient and compared to their respective RRAT scores. The ASA score and RRAT score were significantly correlated (Spearman Rho = 0.324, P<0.001, Figure 1). Thirty-day readmission rates across the system decreased from 3.7% in the year prior to the implementation of RRAT to 2.61% (p<0.05) after RRAT implementation.

## Discussion

Our study demonstrates that the implementation of the RRAT tool across multiple hospitals can lead to a decrease in 30-day readmission rates. We also demonstrated a significant increase in readmission rates with higher RRAT scores. Patients who had scores between 4 and 6 had a 4.27% readmission rate and patients with scores >6 had a 13.64% readmission rate, whereas patients with scores less than 4 only had a readmission rate of 2.35%. One of the primary goals of RRAT implementation in our health system was to ensure there was no bias in offering patients the necessary surgeries and restricting care for high-risk patients. At the inception of the program, the multidisciplinary RRAT team of nurses and physicians was involved in helping develop care plans and pathways to help patients optimize modifiable risk factors. When patients were identified as having risk factors, triggers were made to targeted optimization pathways. A resource list for each category was created and specific recommendations and interventions were proposed. This was given to both the patient and their surgeon. We also sent letters to the surgeon when any patient had an RRAT score over 4 along with a list of recommended interventions prior to any operative intervention. While delaying surgery and optimization was recommended, it was not required and was left to the discretion of the individual surgeon and patient.

The transition from fee-for-payment service models to bundled payment and episode-of-care models has led to increased scrutiny of costs, readmissions, and value-based care. The implementation of these programs has been shown to decrease readmissions, costs, and length of stay in TJA [10]. These various methods range from looking at preoperative patient optimization pathways and intraoperative measures that increase efficiency and decrease cost, to transitioning patients back to home without any complications or readmissions. Bosco et al. analyzed the cost burden of 30-day readmissions following THA and TKA procedures in the Medicare population [11]. The mean cost of unplanned 30-day readmission was $17,103 for primary THA and $13,008 for primary TKA in their analysis. They concluded that hospital cost margins for Medicare THA and TKA are small and any added costs due to readmissions in these narrow margins may make these procedures financially impractical for some hospitals. Phillips et al. analyzed the cost of readmission following primary THA and TKA in a bundled payment model [12]. They concluded that the mean additional cost was $8,588 per readmission. The majority of the patients in their study were readmitted for ‘medical reasons’ (79.1%). Financially, the patients who were readmitted for revision arthroplasty surgery (20.9%) had the highest mean readmission cost ($15,356 per patient). In addition, readmissions for revision surgery had the highest mean post-acute care ($37,207) and overall episode-of-care costs ($52,162).

In an effort to help curtail the costs of readmissions, tools that preoperatively assess and stratify patients that are at high risk for readmission and allow physicians to take appropriate measures to decrease their readmission risks are becoming vital. Hao et al. developed and validated a real-time 30-day hospital readmission risks assessment tool [13]. They were able to validate their readmission tool both retrospectively and prospectively across all diseases, demographics, and payers. By identifying clinical, demographic, and utilization risk factors, their tool provided clinicians with real-time data to help tailor discharge plans to each patient in order to address those risks. Vetter et al. outlined a set of measures at their institution called the preoperative assessment and global optimization (PASS-GO) program for patients undergoing TJA [14]. Their program underscores patient-centered care, shared decision-making, and rigorous process standardization. They used advanced practice providers to collect patient information and stratify the patients into one of three risk categories. The higher-risk patients were then brought in for an in-person, preoperative evaluation and management encounter with an advanced-practice provider or physician (anesthesiologist or internist) depending on how high their risk level was; the patients were then referred to medical subspecialists as deemed necessary for further testing and optimization. Low-risk patients were scheduled for surgery immediately after having a brief telephone interview with an anesthesia nurse; they were then evaluated by an anesthesiologist on the day of surgery.

Kim et al. developed the perioperative orthopedic surgical home (POSH) initiative, which is a surgeon-led screening and optimization pathway targeting the eight modifiable comorbidities as defined by RRAT for patients undergoing TJA [4]. In their study, they included only those patients undergoing unilateral TJA with an RRAT score >3 (higher risk for readmission). The patients were then divided into two groups: patients who were medically optimized by their surgeon using the POSH initiative and those patients who proceeded with surgery without medical optimization (non-POSH). They found that patients in the POSH group with RRAT scores ranging from 3 to 5 had both lower 30-day (1.6% vs 5.3%, P = .03) and 90-day (3.2% vs 7.4%, P < .05) readmission rates when compared to the non-POSH group. Furthermore, only 15.3% of patients in the POSH group were discharged to a post-acute care facility compared to 23.4% of patients in the non-POSH group (P = .01). Ninety-day episode-of-care costs were 14.9% greater among non-POSH Medicare patients and up to 32.6% higher if readmission had occurred. They were able to show that proper preoperative optimization with surgeon involvement can not only lead to better outcomes but can also help decrease readmissions and costs. Similarly, Siracuse et al. developed the readmission after total hip replacement risk (RATHRR) scale by analyzing preoperative risk factor data collected from the New York and California State Inpatient Databases on patients readmitted after primary and revision THA [15]. They were able to reliably explicate readmission variability for 89.1% of the patients in the Florida and Washington State Inpatient Databases validating the application of their readmission scale to other parts of the country.

There were several limitations to our study. The RRAT protocol was launched system-wide between five hospitals. Each hospital had differing resources and protocols for their TJA patients, which can make standardization difficult during the initial implementation period. Moreover, each hospital had different surgeons with variations in their surgical techniques and acute postoperative protocols, which can also contribute to differing outcomes. However, it should be noted that despite the lack of standardization of surgical and postoperative protocols, the overall readmission rate for TJA decreased system-wide. The authors believe that had there been a standardization of the acute postoperative protocols, the difference in readmission rates may be even greater. The implementation of RRAT can be applied across a multitude of hospital settings, from large tertiary care centers to smaller community hospitals. Preoperative screening of patients can also lead to concerns over the restriction of care. One of the fundamental goals of this study was to not restrict care to those with comorbidities and to optimize their medical problems before surgery. Concerns over “cherry-picking” patients or “lemon-dropping” were not lost and why cardiologists, internists, and anesthesiologists being included on the RRAT team to set goals for optimization was imperative to our outcomes. We did not deny surgery for patients with higher scores but did our best to optimize their comorbidities and lower their overall risk factors when possible.

## Conclusions

As hospitals look to transition to performance-based reimbursement for care and to cut unnecessary costs, preemptive intervention to decrease costly readmission rates is vitally important. Using the RRAT helped decrease our health system’s TJA readmission rates. In order to be successful, the RRAT (or any other preoperative optimization plan) requires an active role by the surgeons, anesthesiologists, medical specialists, and hospital system. Participation and teamwork by all departments involved in the perioperative period are important for the common goal of decreasing readmission rates. The study helps further validate the efficacy and clinical usefulness of RRAT across multiple hospitals.
